# The burden of leukemia in the Kingdom of Saudi Arabia: 15 years period (1999–2013)

**DOI:** 10.1186/s12885-019-5897-5

**Published:** 2019-07-17

**Authors:** Amen Bawazir, Nouf Al-Zamel, Abeer Amen, Maaged A. Akiel, Naif M. Alhawiti, Ali Alshehri

**Affiliations:** 1 College of Public Health and Health Informatics, King Saud Bin Abdulaziz University for Health Sciences, Riyadh, Saudi Arabia; 2 King Faisal Specialist Hospital and Research Center, Riyadh, Saudi Arabia; 3 College of Dentistry, Aden University, Aden, Yemen; 4 Department of Clinical Laboratory Sciences, College of Applied Medical Sciences, King Saud Bin Abdulaziz University for Health Sciences, Riyadh, Saudi Arabia; 5 King Abdullah International Medical Research Center (KAIMRC), Riyadh, Saudi Arabia

**Keywords:** Leukemia, Incidence, Cancer, Burden, Saudi Arabia

## Abstract

**Background:**

Leukemia is a malignant neoplasm that arises from hematopoietic cells. The number of leukemia cases has dramatically increased from 297,000 to 437, 033 cases worldwide. As result, the the Saudi Cancer Registry ramked leukemia as the 5th type of cancer cases among both genders in Saudi Arabia. Data on the trend and incidence of leukemnia in Saudi Arabia is lacking. This study aims to report the trend and incidence of leukemia in Saudi Arabia using available data from the Saudi Cancer Registry (SCR), as a population-based cancer registry in the country over a period of 15 years (1999–2013).

**Methods:**

Data of registered leukemia cases between years 1999–2013 were retrieved from the Saudi Council of Health, Saudi Cancer Registry. Data were coded using the International Classification of Diseases for Oncology (ICD-O). Main and essential variables were retrieved such as age, sex, years of incidence, residency, and histopathological type of leukemia.

**Results:**

A total of 8712 cases of leukemia were analyzed in this study, 57.2% were males and 42.8% were females. Around 33.6% of cases were from the central region of Saudi Arabia. The most diagnosed type of leukemia was the Precursor B-cell lymphoblastic leukemia (18.7%), followed by Precursor cell lymphoblastic leukemia, NOS (17.3%) with equal percentage of reported cases between males and females in these subsets.

**Conclusion:**

Ove a period of 15 years, the trend of leukemia showed the likelihood of increase in rate particularly in males with highest incidence reported from the central region of Saudi Arabia which needs more investigation. Resources for diagnosis and treatment should be planned with more orientation toward the accurate diagnosis of leukemia to minimize the number of “none specific diagnosis”.

## Background

Leukemia is a malignant neoplasm of hematopoietic origin, characterized by diffuse replacement of bone marrow and peripheral blood with neoplastic cells [1]. Although, many subtypes of leukemia were known, four main subtypes were frequently seen in diagnosis such as: Acute Myeloid Leukemia (AML), Chronic Myeloid Leukemia (CML), Acute Lymphoblastic Leukemia (ALL) and Chronic Lymphocytic Leukemia (CLL). Globally, between 1990 to 2018, the number of leukemia cases markedly increased from 297,000 to 437, 033 [2]. Thus, according to GLOBOCAN report in 2018, leukemia was ranked the 13th among cancers worldwide, while leukemia deaths increased by 16.5% in the same year. Despite the increased incidence of leukemia over the time, causes of leukemia are still not clear. Both genetic and environmental risk factors such as exposure to ionizing radiation, infection, or chemical substances contribute heavily to the development of leukemia [3]. Consequently, such wide range of risk factors affects prognosis, treatment plans and overall survival [4].

According to the reported data from the GLOBOCAN for region of Middle-East and Northern Africa (MENA), the estimated crude incidence is 5.3 per 100.000 among male population and 4.0 per 100,000 females [5]. Moreover, Gulf Cooperation Council report on cancer, ranked leukemia as the 4th among the most common cancers in the area [6].

The national healthy survey reported that increased prevalence of leukemia lesions among Saudi population is alarming for the healthcare service. This is because of serious complications of leukemia. In 2017, the Saudi Cancer Registry, stated that leukemia was ranked 5th among cancers in both genders of all ages in the Saudi population. The overall prevalence of leukemia was 7.6% in males and 4.4% in females in saudi population [7]. When looking at the age group of older than 14 years of age, leukemia ranked in the top seventh (3.7%), while it ranked the first (38.8%) among Saudi children of less than 14 years of age, with higher rates in males compared to females (59.6% vs. 40.9%).

In this study, we aim to define the burden patterns and trends of leukemia over the period 1999–2013 within Saudi population using the SCR. In addition, the study is aimed to identify the most common types of leukemia in different ages, genders, and regions in the Saudi population. This descriptive study would provide helpful information to decision makers to better understand the demographics of leukemia in Saudi Arabia to help identify patients groups with highest burden to the healthcare system.

## Methods

### Study design and setting

**Fig. 1:**
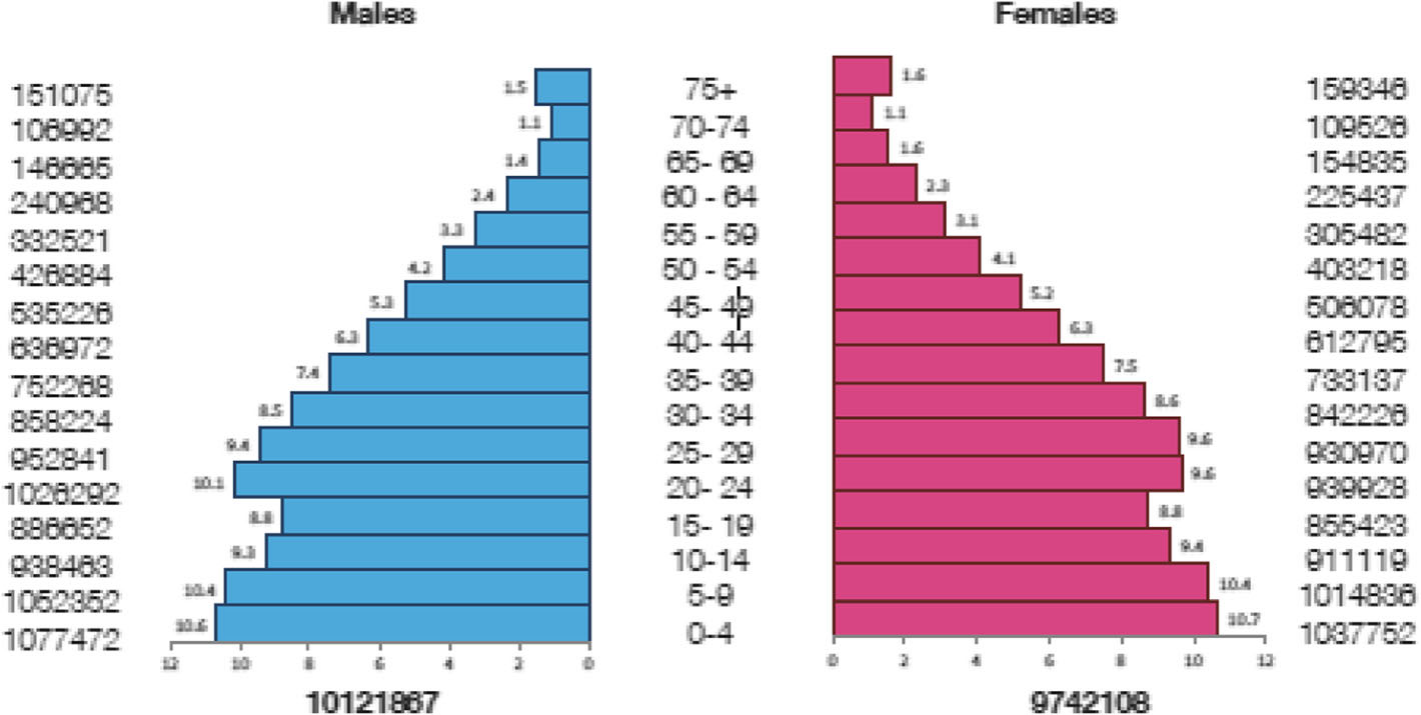
Population pyramids of Saudis (%) by gender and age group, 2015. Male  Female

A retrospective descriptive epidemiological analysis of all Saudi leukemia cases was retrieved from the SCR between January 1st, 1999 and 31st of December 2013 (15 years period). Saudi Arabia is a vast country extending over four-fifths of the Arabian Peninsula. It is approximately 2,149,700 km^2^ in area. According to the national estimation in the year 2015, Saudi citizens were around 19,863,975 with dominant of young population as seen in the population pyramid (Fig. 1) [8].

Saudi Cancer Registry is a population-based registry of all cancer cases registered since the year 1992. The registry is housed in the Department of National Registries in the Saudi Health Council under the admiration of Saudi Ministry of Health (MOH). The SCR strives for full access to cancer data from all Ministry of Health and other governmental and private hospitals, as well as clinics and laboratories throughout the following administrative areas of the country: Riyadh, Qassim, Hail, Dammam, AL Ahsa, Hafr Al-Batin, Jeddah, Makkah, Taif, Qunfudhah, Abha, Asir, Baha, Najran, Jazan, Bisha, Madinah, Tabuk, Jouf and Northern regions of the Kingdom. Thus, the SCR covers all regions of Saudi Arabia as mentioned above. Cancer data are abstracted from patients’ medical records, based on clinical and/or histopathological diagnosis, by SCR certified tumor registrar [9]. A ministerial decree has categorized cancer as a mandatory notifiable disease. This ensures the opportunity for comprehensive data collection from all over the stated regions and health care services. Although the registry reported Non-Saudi cancer cases, this work is reporting only for Saudi nationality.

### Study subjects and data collection

#### Inclusion criteria

All Saudi patients recorded in the registry with diagnosis of leukemia, both genders, in different age groups between the periods of 1st January 1999 to 31st December 2013 were included in this study.

#### Exclusion criteria

Non-Saudi patients with leukemia and registered cases missing principle data, such as age, topography, or sex identification were excluded in this study [11 cases (0.126%).

Requested data from the SCR were serial number, gender, age, address, nationality. Moreover, data related to tumor details like diagnosis date, primary site, histology, behavior, grade, stage, basis of diagnosis and status of the patients (death, alive) were also collected. The primary site (topography) and histology (morphology) of the malignancies are identified and coded according to the International Classification of Diseases for Oncology 3rd Edition (ICD-O-3), published by the World Health Organization (WHO), 2000, while those reported initially with ICD-O-3 codes are converted to ICD-10 for analysis purposes [10, 11].

### Statistical analysis

Data were received from the SCR in a flash disc in form of Excel Sheet and arranged in column based on the required variables as mentioned above. Data were reviewed for incompleteness or missing entries and then entered into the computer package using IBM SPSS Software (Statistical Package for the Social Sciences, version 20, Chicago, Ill, USA). Continous variables such as age were summarized and reported with mean and standard deviation (±SD). Categorical variables such as gender, types of leukemia included Precursor B-cell lymphoblastic leukemia, Precursor cell lymphoblastic leukemia, NOS, Chronic myeloid leukemia, NOS, Acute myeloid leukemia, NOS, B-cell chronic lymphocytic leukemia/small lymphocytic lymphoma, Precursor T-cell lymphoblastic leukemia, Acute promyelocytic leukemia, t(15;17)(q22;q11–12), Acute monocytic leukemia, Acute leukemia, NOS, Acute myelomonocytic leukemia, Acute myeloid leukemia with maturation, Acute myeloid leukemia without maturation, Chronic myelogenous leukemia, BCR/ABL positive were categorized". and presented in frequency and percentages. A two-sided chi-square test was used for independence categorical data with a *P*-value of (< 0.05) considered significant in this study. Estimation of the population based on sex was obtained annually from the Annual year Book from the General Authority of Statistics/Kingdom of Saudi Arabia [12]. The incidence rate was calculated based on mid-time of the total population denominators in the country for the period 1999–2013. The World Standard Population was also used for direct standardization to calculate age-standardized rates per 100,000 populations. The age category classification of the IARC was used, [5] based on age groups of five-year interval (0–4 up to 70–74) patients older than 75 were noted as ≥75. Moreover, the Age-standardized rate is a summary measure of a rate that a population would have if it had a standard age structure. The World Standard Population was used to calculate this stage. The calculated incidence is known as the World Standardized Incidence Rate and the rate is expressed per 100,000 populations.

### Ethics approval and consent to participate

No need for informed consent because the used data was aggregation of secondary data. However, all the data retrieved from the SCR were anonymus just with coded number and without any link to patient’s identity. The proposal of this study was approved by the research committee in the College of Public Health and Health Informatics at the King Saud Bin Abdulaziz University for Health Sciences (KSAU-HS) and thenafter reviewed and approval by the research committee in the King Abdullah International Medical Research Centre (KAIMRC) in the ministry of National Guard Health Affair, Riyadh, Kingdom of Saudi Arabia under the reference (SP 17/030/lR).

## Results

### Sociodemographic characteristics

A total of 8,712 leukemia cases were documented in the SCR during the study period 1999–2013.The mean age was 30.4 (±SD 25.6), 4,984 (57.2%) males and 3,728 (42.8%) were females. The Central region reported the highest rates (33.6%) among other Saudi administrative regions, while Madinah region reported the lowest (14.3%) (Table 1).

**Table 1:** Sociodemographic characteristics of the study population (1999–2013)

Variables		No.	%
Participant Characteristics			
Mean age (±standard deviation)	30.4 (±25.6)		
Gender	Male	4,984	57.2
	Female	3,728	42.8
Regions	Central	2,908	33.6
	Western	1,860	21.5
	Eastern	1,407	16.2
	Southern	1,254	14.5
	Madinah	1,238	14.3
Total	–	8,712	100

.

Five years age -adjusted incidence rate (ASR) by gender of Saudi leukemia cases (1999–2013) was analyzed. In general, the overall ASR was 5.3/100,000 population, however, males showed higher incidence than females (5.9 vs. 4.6 per 100,000 population). According to age groups, the overall highest ASR of leukemia was reported in the youngest age group of 0–4 years old (1.0/100,000 population), followed descendentely by each age group, while the lowest ASR was seen in the middel-age group of 25–39 years old (2.0/100,000 population). Similar trend was found when comparing leukemia ASR by gender (Table 2).

**Table 2:** Five years age -adjusted rate by gender of Saudi leukemia cases (1999–2013)

Age	Males	Females	Total
0–5	1.1	0.9	1.0
5–9	0.6	0.4	0.5
10–14	0.4	0.3	0.3
15–19	0.5	0.3	0.4
20–24	0.3	0.2	0.3
25–29	0.3	0.2	0.2
30–34	0.2	0.2	0.2
35–39	0.2	0.2	0.2
40–44	0.2	0.3	0.3
45–49	0.3	0.3	0.3
50–54	0.3	0.3	0.3
55–59	0.3	0.3	0.3
60–64	0.4	0.3	0.4
65–69	0.4	0.3	0.3
70–74	0.3	0.2	0.2
≥75	0.4	0.2	0.3
All age	5.9	4.6	5.3

### Trend of leukemia in Saudi Arabia overtime

When analyzed the trend of leukemia over the period of 15 years in Saudi Arabia, it shows a steady increase with higher rate of recently reported cases since the year 1999 up to 2013 (5.2 and 7.9%) with similarity in both genders, however the observed differences, was not statistically significant. Leukemia trend was also varied among different administrative regions, with highest rates in children (younger than 5 years of age) among cases reported from Central region of Saudi Arabia (34.2%), followed by adults (15–59 years), and then elder age groups (more than60 years of age) with 33.1, and 33.3%, respectively, but did not showed statistical significant differences (Table 3).

**Table 3:** The trend of Saudi leukemia cases by years and regions (1999–2013)

Variables	Male		Female		Total		p- value
Years	No.	%	No.	%	No.	%	(males/females)
1999	254	5.1	195	5.2	449	5.2	0.550
2000	259	5.2	216	5.8	475	5.5	
2001	300	6.0	193	5.2	493	5.7	
2002	275	5.5	209	5.6	484	5.6	
2003	276	5.5	208	5.6	484	5.6	
2004	291	5.8	230	6.2	521	6.0	
2005	296	5.9	206	5.5	502	5.8	
2006	328	6.6	225	6.0	553	6.3	
2007	355	7.1	253	6.8	608	7.0	
2008	364	7.3	268	7.2	632	7.3	
2009	367	7.4	287	7.7	654	7.5	
2010	391	7.8	276	7.4	667	7.7	
2011	394	7.9	344	9.2	738	8.5	
2012	432	8.7	335	9.0	767	8.8	
2013	402	8.1	283	7.6	685	7.9	
Regions							
Central	1,673	33.7	1,235	33.3	2,908	33.6	0.948
Western	1,069	21.6	791	21.3	1,860	21.5	
Eastern	802	16.2	605	16.3	1,407	16.2	
Southern	705	14.2	549	14.8	1,254	14.5	
Madinah	711	14.3	527	14.2	1,238	14.3	

Analysis of age groups of leukemia cases by gender showed no significant differences between the genders in children and adult age groups, however among the elder groups significant differences was found between genders (*p* < 0.001), but not between regions (Table 4).

**Table 4:** Comparison of age groups of the leukemia cases by gender and region

Variables	Children		Adults		Elderly		
	No.	%	No.	%	No.	%	*P* value
Gender							
Male	1,882	56.7	2,090	55.0	1,012	63.4	< 0.001
female	1,436	43.3	1,709	45.0	583	36.6	
Regions							
Central	1,127	34.2	1,252	33.1	529	33.3	0.612
Western	704	21.3	833	22.0	323	20.3	
Eastern	540	16.4	611	16.2	256	16.1	
Madinah	442	13.4	552	14.6	244	15.4	
Southern	486	14.7	532	14.1	236	14.9	

### Types of leukemia by gender

According to the morphology classified of leukemia cases, 15 types of leukemia were enumerated in this study. The highest reported type was Precursor B-cell lymphoblastic leukemia (18.7%) with equal percentage in both males and females, followed by precursor cell lymphoblastic leukemia not specify [(NOS) (17.3%)], and then chronic myeloid leukemia, NOS (13.0%). The lowest reported type was acute myeloid leukemia without maturation and chronic myelogenous leukemia, BCR/ABL positive (1.2% for each). Chi-square test showed strong significant differences between males and females according to type of leukemia (*p*-value < 0.001) (Table 5).

**Table 5:** Leukemia subtypes by gender (1999–2013)*

Variables	Male		Female		Total	
Morphology	No.	%	No.	%	No.	%
Precursor B-cell lymphoblastic leukemia	934	18.7	696	18.7	1,630	18.7
Precursor cell lymphoblastic leukemia, NOS	875	17.6	630	16.9	1,505	17.3
Chronic myeloid leukemia, NOS	575	11.5	556	14.9	1,131	13.0
Acute myeloid leukemia, NOS	554	11.1	486	13.0	1,040	11.9
B-cell chronic lymphocytic leukemia/small lymphocytic lymphoma	499	10.0	251	6.7	750	8.6
Precursor T-cell lymphoblastic leukemia	256	5.1	67	1.8	323	3.7
Acute promyelocytic leukemia, t(15;17)(q22;q11–12)	155	3.1	160	4.3	315	3.6
Acute monocytic leukemia	125	2.5	109	2.9	234	2.7
Leukemia, NOS	129	2.6	89	2.4	218	2.5
Acute leukemia, NOS	99	2.0	99	2.7	198	2.3
Acute myelomonocytic leukemia	94	1.9	105	2.8	199	2.3
Acute myeloid leukemia with maturation	83	1.7	83	2.2	166	1.9
Acute myeloid leukemia without maturation	50	1.0	53	1.4	103	1.2
Chronic myelogenous leukemia, BCR/ABL positive	59	1.2	48	1.3	107	1.2
All others	497	10.0	296	7.9	793	9.1

*p value of < 0.001

### Distribution of leukemia subtypes by region

Significant differences and variation in the rate of leukemia were found between the subtypes of leukemia and regions of the kingdom (*p* < 0.01). While the Central region had the highest number of precursor B-cell lymphoblastic leukemia cases (21.8%), the western region showed the highest rate in precursor cell lymphoblastic leukemia NOS (21.5%). Also, precursor cell lymphoblastic leukemia, NOS had the highest number in Madinah region (19.1%), followed by precursor B-cell lymphoblastic leukemia (18.3%). The lowest reported subtypes were chronic myelogenous leukemia, BCR/ABL positive (0.6%) in the southern region and also leukemia, NOS (0.7%) in the Central region of the country. In Eastern region the acute myeloid leukemia was reported with higher rate in comparison to other regions (13.1%) (Table 6).

**Table 6:** The distribution of leukemia subtypes among the regions (1999–2013)*

Regions	Central		Eastern		Madinah		Southern		Western	
Morphology	No.	%	No.	%	No.	%	No.	%	No.	%
Precursor B-cell lymphoblastic leukemia	635	21.8	237	16.8	226	18.3	238	19.0	288	15.5
Precursor cell lymphoblastic leukemia, NOS	409	14.1	240	17.1	237	19.1	215	17.1	400	21.5
Chronic myeloid leukemia, NOS	375	12.9	177	12.6	152	12.3	173	13.8	251	13.5
Acute myeloid leukemia, NOS	334	11.5	184	13.1	145	11.7	160	12.8	204	11.0
B-cell chronic lymphocytic leukemia/small lymphocytic lymphoma	275	9.5	111	7.9	89	7.2	113	9.0	155	8.3
Precursor T-cell lymphoblastic leukemia	129	4.4	38	2.7	34	2.7	39	3.1	82	4.4
Acute leukemia, NOS	25	0.9	20	1.4	46	3.7	43	3.4	62	3.3
Acute monocytic leukemia	98	3.4	46	3.3	21	1.7	24	1.9	44	2.4
Acute promyelocytic leukemia, t(15;17)(q22;q11–12)	114	3.9	63	4.5	40	3.2	48	3.8	50	2.7
Leukemia, NOS	20	0.7	45	3.2	81	6.0	25	2.0	45	2.4
Acute myelomonocytic leukemia	74	2.5	30	2.1	26	2.1	27	2.2	42	2.3
Acute myeloid leukemia with maturation	72	2.5	24	1.7	21	1.7	20	1.6	29	1.6
Chronic myelogenous leukemia, BCR/ABL positive	31	1.1	19	1.4	9	0.7	8	0.6	40	2.2
Acute myeloid leukemia without maturation	42	1.4	15	1.1	16	1.3	14	1.1	16	0.9
All others	275	9.5	158	11.2	95	7.7	107	8.5	152	8.2

*p value of < 0.001

### Diagnostic and prognostics characteristics of leukemia among Saudi regions (1999–2013)

A total of 8712 cases of leukemia in all Saudi regions were diagnosed by blood and bone marrow examinations, followed by other types of diagnostic methods (*p* < 0.01). According to the Immunophenotyping diagnostic procedure, the B-cell leukemia was highest in all the regions (84.3%), followed by T-cell (14.7%). Rates of cancer related deaths were so close to the overall causes of death in the study population, which ranged from 27.1% in the Western region to 30.0% in the Central region of Saudi Arabia (Table 7).

**Table 7:** Diagnostic and prognostic characteristics of leukemia cases among Saudi regions (1999–2013)

Regions	Central		Eastern		Madinah		Southern		Western				P value
Immunophenotyping	No.	%	No.	%	No.	%	No.	%	No.	%	No.	%	
B-cell	1,105	83.7	487	84.4	411	88.2	419	84.8	572	82.5	3008	84.3	0.297
T-cell	206	15.6	85	14.7	53	11.4	67	13.6	110	15.9	523	14.7	
Grade I -IV	4	0.3	2	0.3	1	0.2	2	0.4	2	0.3	12	0.3	
Killer cell	2	0.2	1	0.2	1	0.2	1	0.2	2	0.3	7	0.2	
Null cell	3	0.2	2	0.3	0	0.0	5	1.0	7	1.0	17	0.5	
Basis of Diagnosis													
Histology of primary	2,080	71.8	1,006	72.5	704	58.4	815	65.4	1,172	63.5	5805	67.3	0.001
Death Certificate Only	36	1.2	30	2.2	74	6.1	38	3.0	47	2.5	228	2.6	
Others	780	26.9	351	25.3	427	35.4	394	31.6	628	34.0	2594	30.1	
Causes of Death													
Cancer	873	30.0	372	26.4	359	29.0	355	28.3	504	27.1	2477	28.4	0.085
Others	2,035	70.0	1,035	73.6	879	71.0	899	71.7	1,356	72.9	6235	71.6	

### Distribution of leukemia subtypes in the three age groups

Significant differences were found between the subtypes of leukemia in different age groups (children, adults, and elders). However, the children (≤14 years old) had the highest percentage of precurso B-cell lymphoblastic leukemia cases (36.6%), and precursor cell lymphoblastic leukemia-NOS s (31.4%), while cases of B-cell chronic lymphocytic leukemia/small lymphocytic lymphoma and chronic myelogenous leukemia (BCR/ABL positive) were the lowest percentage (0.1%). In adults (15–59 years old), chronic myeloid leukemia-NOS was reported as the most common subtypes of leukemia (23.0%), followed by Acute myeloid leukemia-NOS (14.6%). The lowest percentage of cases of acute myeloid leukemia in adults without maturation was (1.6%). Among the elderly (60–76 years old), the highest percentage of leukemia was B-cell chronic lymphocytic leukemia/small lymphocytic lymphoma (32.5%), followed by acute myeloid leukemia-NOS (14.8%), while the lowest percentage among the elderly group was Precursor T-cell lymphoblastic leukemia (0.1%) (Table 8).

**Table 8:** The distribution of leukemia subtypes in three age categories (1999–2013)

Variables	Children		Adults		Elderly		
Morphology	No.	%	No.	%	No.	%	*P* value
Precursor B-cell lymphoblastic leukemia	1,215	36.6	388	10.2	27	1.7	0.001
Precursor cell lymphoblastic leukemia, NOS	1,043	31.4	404	10.6	58	3.6	
Precursor T-cell lymphoblastic leukemia	177	5.3	144	3.8	2	0.1	
Acute myeloid leukemia, NOS	250	7.5	554	14.6	236	14.8	
Acute leukemia, NOS	68	2.0	78	2.1	52	3.3	
Acute monocytic leukemia	55	1.7	126	3.3	53	3.3	
Leukemia, NOS	34	1.0	84	2.2	100	6.3	
Acute myelomonocytic leukemia	37	1.1	127	3.3	35	2.2	
Acute myeloid leukemia with maturation	45	1.4	91	2.4	30	1.9	
Acute myeloid leukemia without maturation	18	0.5	60	1.6	25	1.6	
Acute promyelocytic leukemia, t(15;17)(q22;q11–12)	61	1.8	220	5.8	34	2.1	
B-cell chronic lymphocytic leukemia/small lymphocytic lymphoma	2	0.1	229	6.0	519	32.5	
Chronic myelogenous leukemia, BCR/ABL positive	4	0.1	86	2.3	17	1.1	
Chronic myeloid leukemia, NOS	59	1.8	875	23.0	197	12.4	
All others	250	7.5	333	8.8	210	13.2	

### Incidence of leukemia

**Fig. 2:**
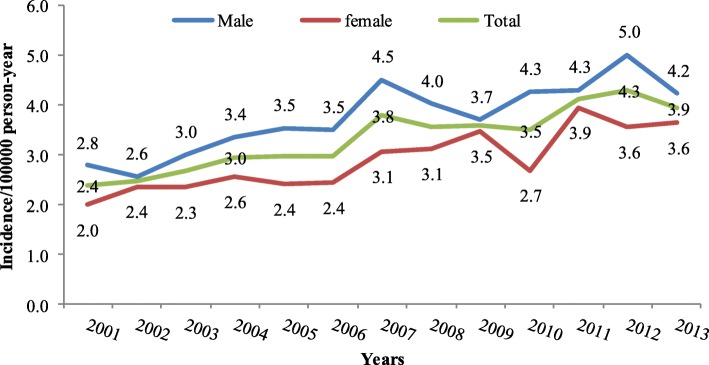
The overall incidence of leukemia over the period 2001-2013 by gender

**Fig. 3:**
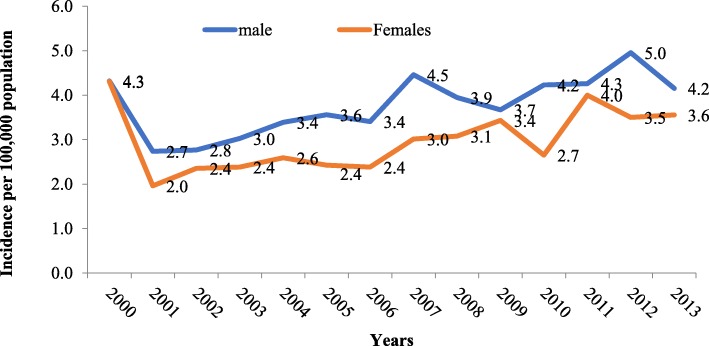
Leukemia incidence in children for the period 2001-2013 by gender

**Fig. 4:**
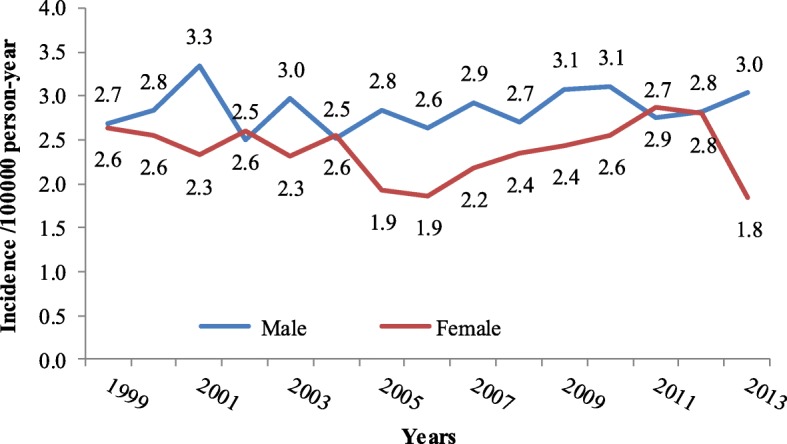
Leukemia incidence in adults for the period 2001-2013 by gender

**Fig. 5:**
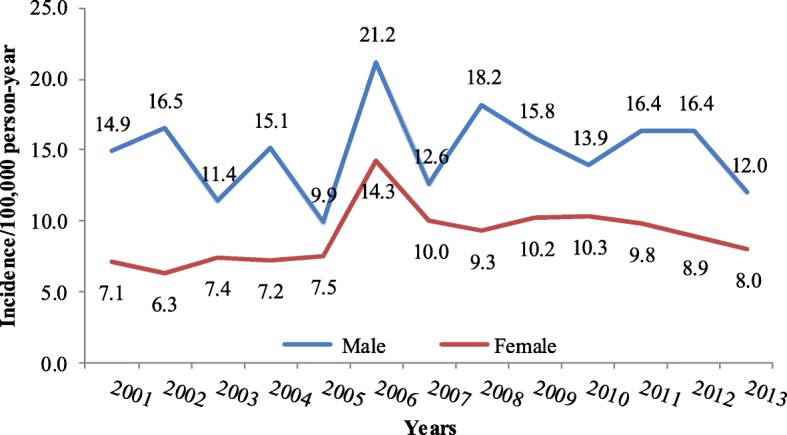
Leukemia incidence in elderly for the period 2001-2013 by gender

The overall incidence of leukemia during the period (1999–2013) has steadily increased among both genders (Fig. 2). However, it is more in males than females. In 2012, the peak among males was reported in the as 5.5 per 100,000 person per year in 2013, while in females it was reported as 4.3 per 100,000 person per year in the year 2011, as shown in Fig. 2**.** This increase in 2012 was also observed in young males of less than14 years of age, Fig. 3. The peak of incidence in young males was 4.0 per 100,000 people per year, while it was 3.0 for young females (Fig. 3). In adults of more than 14 years of age, both males and females shown an incidence of 2.7 per 100,000 populations per year (year 1999), while females dropped to 1.8 per 100,000 per year in 2013 (Fig. 4)**.** The overall incidence during the period (2001–2013) among the elderly age group (more than 60 years of age-76). Both genders showed a steadily increased of leukemia with more trend in males than females, Fig. 5. For example, the peak was reported as the highest among male cases in the year 2006 (21.2 per 100,000 person-year), while highest incidence rate was found in the year 2006 in females (14.3 per 100,000 person-year) (Fig. 5).

## Discussion

Leukemia is considered as an important public health problem in Saudi Arabia for it is impact in causing deaths among young people. In this country, around one fourth of the population are children less than 14 years old, highlighting the huge burden of such malignant blood disease. Therefore, the reported mean age in this study was 30.4 years from the total included patients in this study (8712) with higher rate among males than females (57.2% vs. 42.8%). The predominance of leukemia cases were in the age group (0–4 years), followed by the age group (5–9 years). In general, leukemia is considered one of the most kinds of cancer impacted on children, and statistical studies reported that the incidence of leukemia cases of children has higher rates over the globe. However, the occurance of leukemia in the Saudi population was low when compared to developed countries such as United Kingdom, United States, Australia, Canada and Germany. [2] In the United Kingdom for example, Leukemia accounts of 34% of all malignancies in children aged under 15 years. A recent study from Piedmont reported that leukemia was the first cancer among children and adolescents in the years from 1967 to 2011, with a peak age was at 1–4 years (75.9%), similar to our findings [13]. Other study from Yemen (Aden Cancer Registry) stated that leukemia was of higher rate among boys and girls < 15 years old (34.2 and 38.3%, respectively), as well as more in men (10.5%) compared to women (7.6%) [14].

Worldwide, cancer incidence in children varies from 10 to 18 per 100,000 children under 15 years of age [15]. The cancer incidence in KSA increased throughout the years like other developing countries that could be due to the improvement of health care facilities, diagnostic procedures, and easy referral system for further diagnosis and treatment to well recognizable tertiary hospitals and oncology specialized centres in the main cities. Other factor for the notified increase of cancer cases in Saudi population was the remarkable improve in wealth, rapid transitional socioeconomic changes and lifestyle modifications, and thus increase in life expectancy at birth, contribute to the increase of cancer incidence in the country [6, 16]. Development of National Cancer Registry and improvement of registration practices with certified tumour registrars could be considered as additional factors lead to increase in the reporting of more cancer cases during the last years in the country. During the study period, the number of the population in Saudi Arabia have growing by 50% of the population in 1999 compared to the year 2013. This could also explain the increasing trend of leukemia incidence in the country [17].

The current study showed a variation in the distribution of the leukemic cases among Saudi population over the different regions of Saudi Arabian Kingdom. For example, the most reported cases were from the Central region of Saudi Arabia (33.6%), while the lowest rate was reported from Madinah region (14.3%). This could be explained as the fact that the Central region has several mega medical centers such as King Abdulaziz Medical City, King Faisal Specilist Hospital and Research Center, and King Fahd Medical City, all are considered as big referal hospitals that allowing the referr of cases from other regions to the central one. In addition, Riyadh as a capital of the Kingdome, accounte over 7 million people which is around 22% of the population of the whole country [8].

The present study showed that a patterned increase in the overall incidence of leukemia cases during the period (1999–2013) with higher incidence in males compared to females. Similar findings were reported by Jung et al., where the incidence of leukemia in South Korea raised from 4.7/100.000 in the year 1999 to 5/100,000 population in the year 2009 [18]. According to age groups, our findings showed less likely trend among adults those from 15 years through 60 years old in both genders. The current study is consistent with previous studies, which showed that elderly people over 60 year old has high risk for leukemic progression that could be due to immune system dysfunction or idiopathic causes. For example, in 2013, the incidence of leukemia cases in elderly patients (60–99 year old) was 12.0 (male) and 8.0 (female) per 100,000 person (Fig. 4) compared with adult patients (> 14–59 year old) was 3.0 (male) and 1.8 (female) per 100,000 person (Fig. 3). The trend of leukemia cases over the study period have increased from 5.2% in 1999 to 7.9% in 2013, with no significant differences between males and females. Although, the relation of leukemia and cause is of great important in the public health field, this issue is not yet studied extensivly in the country.The Kingdom of Saudi Arabia has went through a massive economic evolution after its entery in the petroluim industry, moreover, as a developing country, leukemia incidence has increased over decade due to several factors, such as:population growth, ageing, western life adoption, role of environmetal factors, genetic risk factors and mony others. Both pre-natal and post-natal exposure to ionizing radiation (particularly X rays) can cause leukemia in children [19]. Pre-natal exposure to X rays has been greatly reduced with the adoption of ultrasound for screening in pregnant women. Several studies link pesticide exposure by both parents and children to leukemia. The pattern of disease suggests that some damage to chromosomes may occur before the child is born [20]. Children born to parents employed in certain occupations that have chemical exposures are more likely to have leukemia [21]. Chemicals, specifically including benzene, have been shown to cause leukemia in adults.

Among Saudi regions, the two most common leukemia subtypes were Precursor B-cell lymphoblastic leukemia followed by Precursor cell lymphoblastic leukemia, NOS. Moreover, the top three most common leukemia subtypes in both genders were: Precursor B-cell lymphoblastic leukemia, followed by Precursor cell lymphoblastic leukemia, NOS and Chronic myeloid leukemia, NOS. This trend in the subtypes of leukemia could also be found in different countries in the Gulf region like United Arab Emirates, Kuwait and Oman [6].

Limitations: Hence secondary data were used in this study, limitations are present due to the used method in data analysis. Moreover, the collection of data was not for the purpose of investigating the burden of leukemia in the Kingdom of Saudi Arabia.

There were some inaccurate information in the data from SCR such as mistakes in age, nationality, and topography. For example, SCR report that the majority of leukemia cases were metastized where the leukemia is diseases of blood and no metastasis should be reported. In addition, The SCR did not register the demographic information of the patient such as marital status and occupation which could be risk factors for specific cancer.

## Conclusion

Over the 15 years period, the trend of leukemia showed the likelihood of increase in rate particularly in males with highest incidence reported from the central region of Saudi Arabia which needs more investigation if cases were really from this region or focused around the capital Riyadh. According to the type of leukemia among the Saudi population, resources for diagnosis and treatment should be planned with more orientation toward the accurate diagnosis. As the “not specific diagnosis” is common among the reported data, more training and facilities should be provided to the health care facilities to bring a good early diagnosis and accordingly appropriate therapy with possible better standard of cure. Findings from the analyzed data based on cancer registries all over the country, still showed significant limitation in reporting standardized data on leukemia from the health care settings. To correct this part and bringing clean and clear data, training is very important to prepare certified tumor registrar over the country regions.

## Data Availability

The datasets used and/or analysed during the current study are available from the corresponding author on reasonable request.

## Abbreviations

ALL: Acute Lymphoblastic Leukemia
AML: Acute Myeloid Leukemia
CLL: Chronic Lymphocytic Leukemia
CML: Chronic Myeloid Leukemia
ICD-O: International Classification of Diseases for Oncology
KAIMRC: King Abdullah International Medical Research Centre
KSAU-HS: King Saud Bin Abdulaziz University for Health Sciences
MENA: Middle-East and Northern Africa
MOH: Ministry of Health
SCR: Saudi Cancer Registry
